# Mechanisms of Cell Adhesion Molecules in Endocrine-Related Cancers: A Concise Outlook

**DOI:** 10.3389/fendo.2022.865436

**Published:** 2022-04-07

**Authors:** Yongsheng Ruan, Libai Chen, Danfeng Xie, Tingting Luo, Yiqi Xu, Tao Ye, Xiaona Chen, Xiaoqin Feng, Xuedong Wu

**Affiliations:** ^1^ Department of Pediatrics, Nanfang Hospital, Southern Medical University, Guangzhou, China; ^2^ Department of Endocrinology, Affiliated Baoan Hospital of Shenzhen, Southern Medical University, Shenzhen, China

**Keywords:** cell adhesion-mediated drug resistance, endocrine-related cancers, tumor microenvironment, cell adhesion molecules, chemoresistance

## Abstract

Chemotherapy is a critical treatment for endocrine-related cancers; however, chemoresistance and disease recurrence remain a challenge. The interplay between cancer cells and the tumor microenvironment *via* cell adhesion molecules (CAMs) promotes drug resistance, known as cell adhesion-mediated drug resistance (CAM-DR). CAMs are cell surface molecules that facilitate cell-to-cell or cell-to-extracellular matrix binding. CAMs exert an adhesion effect and trigger intracellular signaling that regulates cancer cell stemness maintenance, survival, proliferation, metastasis, epithelial–mesenchymal transition, and drug resistance. To understand these mechanisms, this review focuses on the role of CD44, cadherins, selectins, and integrins in CAM-DR in endocrine-related cancers.

## Introduction

Multidrug resistance and disease recurrence are challenging in the treatment of endocrine-related cancers, including breast, ovarian, prostate, pancreatic, and thyroid cancers. Breast cancer is one of the most common cancers in women worldwide and is the leading cause of cancer-related deaths (1). The 5-year survival rate of patients with metastatic breast cancer is approximately 26% (2). The ER/PR/HER2-negative subtype, known as triple-negative breast cancer (TNBC), accounts for approximately 10%–15% of all breast cancers and constitutes the most aggressive breast cancer (3). TNBC has a high metastatic capacity and poor outcome owing to increased recurrence rates, regardless of disease stage and resistance to conventional therapies (4). Ovarian cancer may arise from the histological portions of the ovary, including the epithelium, stroma, or germ cells. Epithelial ovarian cancers are diagnosed at advanced stages and are treated with surgery and chemotherapy (5). Of these, high-grade serous carcinoma arising from the epithelium of the ovary is the most common (5). The clinical management and prognosis of ovarian cancer depend on the cancer stage. Most patients are diagnosed at an advanced stage with widespread peritoneal dissemination and malignant ascites. Since rapidly proliferating tumors compress visceral organs and are only temporarily chemosensitive, ovarian carcinoma is a deadly disease with a cure rate of only 30% (6). Although the incidence rates for pancreatic ductal adenocarcinoma (PDAC) are currently low, about half of the patients have advanced disease at diagnosis, and it is estimated that it will be the second leading cause of cancer-related mortality by 2030. Nearly half of the patients with PDAC have progressive disease at diagnosis, and multiagent chemotherapy regimens only provide a survival benefit of 2–6 months compared with single-agent gemcitabine for advanced patients (7). Metastatic castration-resistant prostate cancer (8) and persistent or recurrent thyroid cancer are highly treatment-refractory and have poor prognoses (9, 10).

Cell adhesion molecules (CAMs) are transmembrane receptor proteins involved in cell-to-cell or cell-to-extracellular matrix (ECM) binding (11). Based on their structures, CAMs can be classified into four major groups: integrins, cadherins, selectins, and the immunoglobulin superfamily CAM (IgCAM) (12, 13). CD44 is an essential cell surface glycoprotein involved in cell-to-cell adhesion (14). Therefore, we considered CD44 as a CAM in this context. CAMs play a role in maintaining cell adhesion and exert crucial cellular functions, such as cell proliferation, survival, migration, and oncogenesis (14–16). Moreover, the cell adhesion-mediated resistance (CAM-DR) restricts the success of cancer therapies and is an enormous obstacle to combat in the clinic (13, 17). This review describes the role of integrins, cadherins, selectins, and CD44 in endocrine-related cancer.

This review attempts to concisely summarize the advances made in this context, emphasizing endocrine-related cancers and the avenues for future progress to target mitotic mechanisms to overcome these dreadful cancers.

## Structures and Function of CAMs

### Integrins

**Figure 1:**
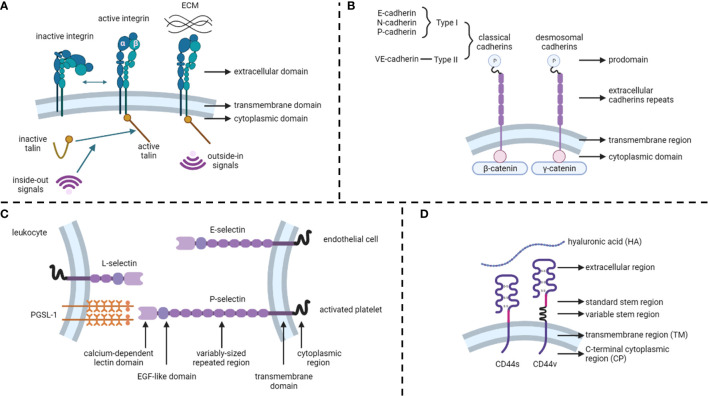
Structures of cell adhesion molecules. **(A)** Integrins. **(B)** Cadherins. **(C)** Selectins. **(D)** CD44. ECM, extracellular matrix.

Integrins are calcium-independent heterodimeric transmembrane proteins with a shared structure of the extracellular, transmembrane, and cytoplasmic domains (**Figure 1A**). To date, 24 functionally distinct integrin heterodimers have comprised 18 integrin α and 8 integrin β subunits (18). Integrins interact with specific ECM ligands *via* bidirectional signaling pathways. Inside-out signals mediate talin binding to integrin β-tails and thus tightly control integrin affinity for ECM ligands. Subsequent ECM binding triggers the recruitment of protein complexes to integrin cytoplasmic tails to facilitate integrin downstream signaling, known as outside-in signaling (19). In addition, integrins can be internalized and recycled, thereby controlling the availability of integrin heterodimers in the plasma membrane (13). Integrins and their ligands play critical roles in cell survival, proliferation, motility, differentiation, and ensuring appropriate cell localization (20, 21).

### Cadherins

Unlike integrins, cadherins are calcium-dependent transmembrane proteins (22) that constitute type I and type II classical cadherins, desmosomal cadherins, proto-cadherins, seven-pass transmembrane cadherins, and FAT and dachsous cadherins (13) (**Figure 1B**). These proteins contain a prodomain (P) immediately after the removal of the signal sequence by proteolysis. Mature classical and desmosomal cadherins have ectodomains composed of five extracellular cadherin repeats, a single transmembrane region, and a cytoplasmic domain that interacts with either β-catenin (classical cadherin) or γ-catenin (desmosomal cadherin) (22). Moreover, E-cadherin (CDH1), N-cadherin, and P-cadherin are classified as type I, whereas VE-cadherins are type II classical cadherins (13). Cadherins regulate cell-to-cell cohesion in all tissues and are indispensable for morphogenesis, maintaining tissue barriers, and regulating tissue remodeling (16, 23).

### Selectins

Selectins (CD62) are single-chain transmembrane glycoproteins that regulate cell-to-cell adhesion *via* carbohydrate-binding in a calcium-dependent manner (24). There are three major types of selectins: L-selectins (primarily expressed on leukocytes), E-selectins (expressed on endothelial cells), and P-selectins (expressed on activated platelets) (25) (**Figure 1C**). The structures of selectins consist of a calcium-dependent lectin domain, an epidermal growth factor (EGF)-like domain, a variably sized repeated region, a transmembrane domain, and a cytoplasmic domain (26). The primary function of selectins is to promote leukocyte rolling along with endothelial cells, which is an initial step in the transmigration of leukocytes through the endothelial barrier (27, 28).

### CD44

The CD44 protein consists of four primary regions: the extracellular region, stem region (standard stem region and variable stem region), transmembrane region (TM), and C-terminal cytoplasmic (CP) region (14) (**Figure 1D**). The CD44 variant (CD44v) differs from the CD44 standard isoform (CD44s) by the insertion or excision of alternatively spliced exons between the N-terminal and C-terminal domains (29). CD44s are distributed in tissues, such as lymphocytes, the central nervous system, lungs, epidermis, pancreas, intestines, kidneys, urinary bladder, and cervix. CD44v is dispersed on keratinocytes, lymphocytes, macrophages, and epithelial cells in the bladder, stomach, and cervix (30). While binding to its primary ligand hyaluronic acid (HA), CD44 has extensive functions, such as cell adhesion, hyaluronate degradation, lymphocyte activation, lymphocyte homing, lymphopoiesis, myelopoiesis, angiogenesis, and release of cytokines (31, 32).

## CAMs in Cancers and Their Microenvironment

CAMs mediate the adhesion of cancer cells not only to each other but also to stromal cells and ECM, namely, the tumor microenvironment (TME). The TME mainly comprises stable nonmalignant cells, including surrounding immune cells, extracellular matrix, fibroblasts, endothelial cells (ECs), blood vessels, and stromal cells (33).

Notably, there is a population of cancer cells named cancer stem cells (CSCs), identified as cells within a tumor with self-renewal capacity and tumorigenic potential (34). In previous studies, CSCs were isolated from various cancers and successfully identified using surface markers, including CD44, CD24, CD133, and CD166 (34, 35), with CD44 being the most common marker (36). For example, a population of cells expressing CD44 high/CD24 low was identified as CSCs in breast cancer (37). Moreover, when CD44 binds to HA, it plays a pivotal role in cancer invasiveness (38). In a previous study, the invasiveness of the human breast cancer cell line MDA-MB-468 was increased by 45% with high-molecular-weight HA and was inhibited by anti-CD44s or HAoligo-6 (39).

CSCs also have characteristics associated with cells undergoing epithelial–mesenchymal transition (EMT) (40). In metastatic epithelial tumors, downregulation of CDH1, compensated by the expression of N-cadherin, is a hallmark of EMT (41). The transforming growth factor-β (TGF-β) pathway plays a central role in activating EMT in several cancers (42, 43). EMT-inducing transcription factors (EMT-TFs), such as Snail, ZEB1, TWIST, and Slug, endow resistance to cisplatin- and oxaliplatin-based chemotherapies in breast, ovarian, and pancreatic cancers and CSC maintenance (44, 45). The invasion and metastasis of cancer implicate the latest steps in malignant progression and most cancer-associated deaths (46). Interestingly, cadherins mediate cancer cell-to-cancer cell adhesion and, subsequently, form collective migration, defined as two or more cells moving together rather than single cancer cell migration (47).

After cancer cells undergo EMT, selectins confer cancer cells that express selectin ligand implantation from blood-borne metastasis. For instance, it was shown that overexpressing c-FOS ovarian cancer cells had lower selectin ligands sLea and sLex and diminished adhesion to E-selectin, thereby reducing metastasis in an intraperitoneal xenograft mouse model (48). Regarding P-selectin, there was a report showing that P-selectin mediated rolling and adhesion of ovarian cancer cells expressing sLex to mesothelial cells (49). Similarly, Gebauer et al. demonstrated that the interaction of E-selectin, P-selectin, and mesothelial cells in pancreatic cancer cells was in a shear stress-dependent manner (50).

This initial interaction between endothelial or mesothelial cells expressing selectins and cancer cells results in the activation of integrins, which mediate firm adhesion to the ECM and stromal cells. Cancer-associated fibroblasts (CAFs) are derived from various cell types including ECs (51), adipocytes (52), pericytes (53), and mesenchymal stromal cells (MSCs) (54). In addition, integrin α11 in breast CAFs originating from MSCs (54) interacts with platelet-derived growth factor receptor beta (PDGFRβ), promotes invasiveness, and produces a matricellular protein (55). Another role of integrins is the maintenance of CSC characteristics, such as CD44 marker. For example, integrin β1 is a CSC marker and its expression is critical for initiating breast cancer tumorigenesis *in vivo*, which is discussed in the following section (56).

CSCs or cancer cells contact the TME through cell-to-cell and cell-to-ECM by CAMs, leading to the initiation of survival and proliferation signaling pathways in cancer cells and secretion of cytokines and growth factors from both cancer cells and stromal cells. Furthermore, this mutual beneficial effect regulates conventional treatment failure, called cell adhesion-mediated drug resistance (CAM-DR), in cancer cells.

## CD44 and Endocrine-Related Cancers

**Figure 2:**
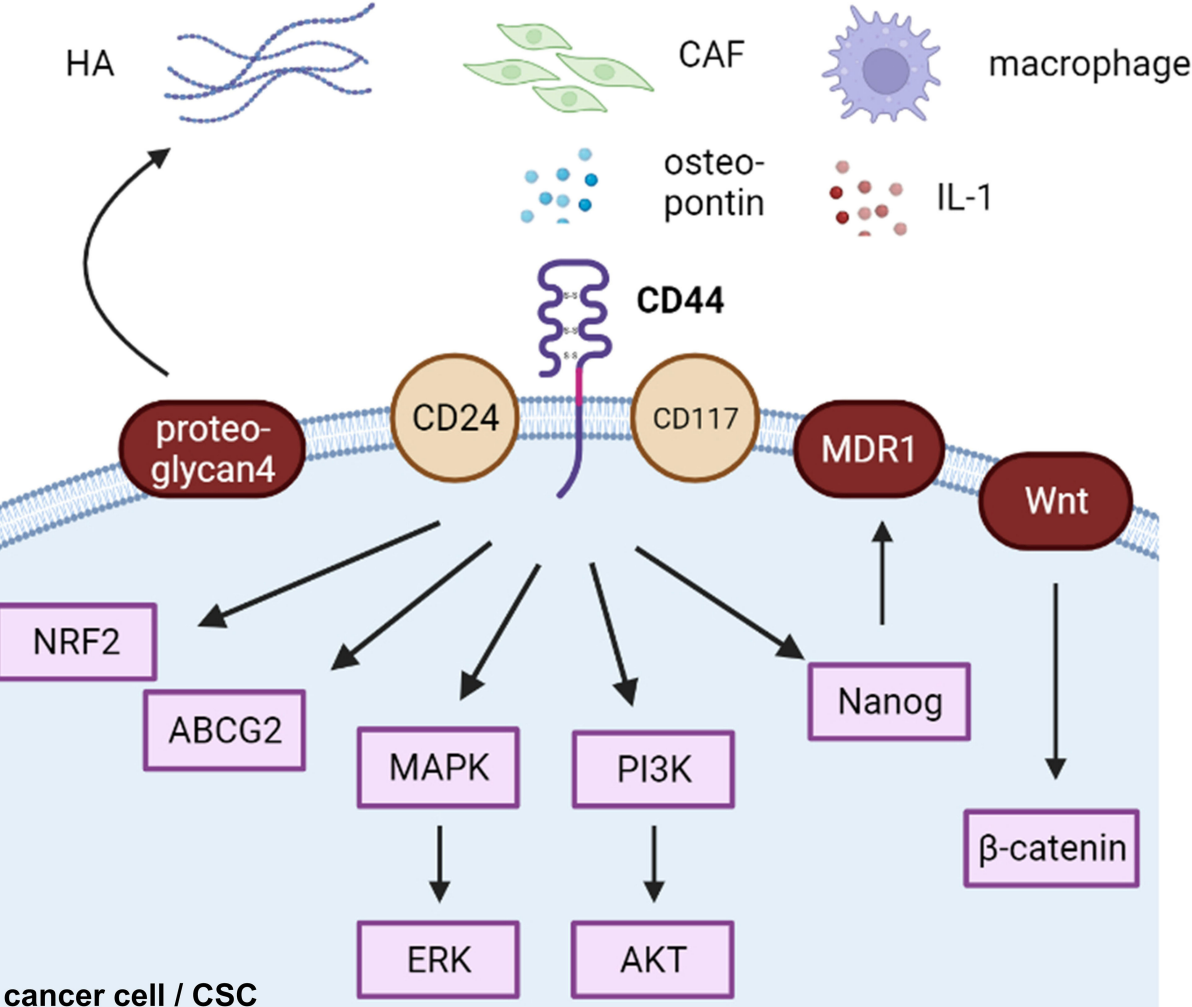
CD44-related cascades in cancer cells. HA, hyaluronic acid; CAF, cancer-associated fibroblast; CSC, cancer stem cell.

CD44-positive expression was predominantly correlated with a high TMN and worse 5-year overall survival in ovarian cancers in a meta-analysis of 18 publications including 2,161 patients (57). CD44 is the most common cancer stem cell receptor, and its overexpression is associated with metastasis, resistance, and tumor recurrence in endocrine-related cancers (38, 58, 59). Although many cells express CD44s on their cell surface, cancerous cells express both CD44s and CD44v on their cell membranes (60). HA interacts with various cell surface receptors, including those involved in intracellular signaling pathways, such as the tyrosine kinase pathway and the hyaluronan-mediated motility receptor CD44, to increase proliferation, survival, and resistance to cancer cells (61) (**Figure 2**).

### CD44 and CSCs

Based on clinical evidence, increased CD44high/CD24low expression was found in a subpopulation of chemoresistant breast CSCs (62). The CD44+/CD117+ subpopulation is known as ovarian CSCs (63). CD44s, rather than CD44v, initiates PDGFRβ/Stat3 signaling to facilitate CSC properties in breast cancer (64). Stress tolerance caused by reactive oxygen species is a characteristic of drug resistance. CD44v enhances protection against ROS and promotes chemoresistance (65). Moreover, long-term CAF-conditioned media facilitates the enrichment of the stemness population of prostate cancer cells and their drug resistance *via* the osteopontin/CD44 axis (66). For example, high CD44 expression results in p62-associated NRF2 activation in breast CSCs. CD44/NRF2 activation contributes to doxorubicin resistance in CD44high breast CSCs (67). Proteoglycan 4, a mucin-like glycoprotein, induces TGF-β and HA expression in breast cancer (68). Therefore, proteoglycan 4 acts as a CD44/HA regulator and promotes metastasis (68). Recently, a study showed that CD44 promoted PD-L1 expression, eliciting an immunosuppressive effect in TNBC using the FACS-assisted shRNA screening method (69). MicroRNA-199a could target CD44, restore chemosensitivity to paclitaxel, cisplatin, and adriamycin, and reduce ABCG2 expression, a multidrug resistance gene in *in vitro* and *in vivo* ovarian cancer models (63).

### HA/CD44/MDR1 Axis

Following the interaction of CD44 with HA, ankyrin binds to multidrug resistance mutation 1 (MDR1) leading to doxorubicin efflux and paclitaxel and chemoresistance in both breast and ovarian cancer cells (70). Moreover, this activation of MDR1 is mediated by CD44/Nanog/Stat-3 signaling (70). Further study demonstrated that inhibitors of apoptosis proteins are also elevated by the CD44/HA/Nanog interaction (71). This process leads to microRNA-21 production and protein programmed cell death 4 reductions (71). HA-CD44-PI3K-ErbB2 constitutes a positive feedback loop in an Akt-independent manner (72). This loop amplifies MDR1 expression and regulates drug resistance in doxorubicin-resistant breast cancer cells MCF-7 (73) and pancreatic cancer cells (73). CD44/HA/MDR1 siRNA NPs have been successfully evaluated in ovarian cancer (74). HA-engineered nanomicelles are taken up by triple-marker positive (CD44+/CD133+/EpCAM+) pancreatic CSCs and induce a cell killing effect (75). HA-modified nano-complexes target CD44 receptor-induced apoptosis of drug-resistant cells *via* mTOR signaling in ovarian cancer SKOV3 cells *in vivo* (76).

### CD44/PI3K, CD44/MAPK, and CD44/Wnt Axis

Increased CD44v6 expression is found in prostate cancer surrounding stromal cells (77) and indicates poor clinical outcomes in pancreatic ductal adenocarcinomas (78). Furthermore, knockdown of CD44v6 suppresses prostate cancer proliferation, reduces EMT, and augments chemosensitivity *via* downregulation of Wnt/β-catenin and PI3K/Akt/mTOR signaling (77). Another study determined that CD44v3 induces MDR1 overexpression, thereby activating P300/β-catenin and NF-κB (60). Calreticulin maintains the breast CSC properties of CD24-/CD44+ and ALDH+ and promotes breast cancer progression *via* Wnt/β-catenin signaling in an HIF-1-dependent manner (79). SRGN is an ECM factor that binds to CD44 in an autocrine manner and acts by triggering MAPK and Wnt/β-catenin signaling in breast CSCs (80). Thus, CD44 is closely regulated by Wnt signaling (33, 81).

Phospho (p)-AKT expression is more likely found in CD44v6-positive breast cancer tissues. Moreover, overexpression of CD44v6 and p-AKT is correlated with poor clinical outcomes (82). Osteopontin/CD44 cascade promotes ovarian cancer cell chemoresistance *via* PI3K/AKT signaling and drug efflux mechanisms (83). Rho GTPase activation has been linked to the prostate cancer cell line PC3 and osteopontin adhesion. Subsequently, surface CD44 and MMP-9 expression increases (84). Furthermore, CD44 has been linked to aggressive pancreatic cancer by the PI3K/AKT or MAPK/ERK pathways in clinical data (85).

Recently, several studies have implied that some interleukins (ILs) also regulate CD44 signaling. IL1β, a pivotal regulator of the systemic inflammatory response, is produced by macrophages in breast cancer. This process is mediated by the breast cancer cell membrane-derived soluble CD44. Therefore, neutralization of CD44 by antibody decreased IL1β production in macrophages and attenuated the growth of primary breast cancer (86). Another similar target strategy using an IL1R2 neutralizing antibody suppressed breast CSC progression (87). IL-6 can generate CD44+ CSCs *via* the induction of EMT in breast cancer cell T47D (88).

## Cadherins and Endocrine-Related Cancers

In recent decades, studies have shown that mechanical EMT mediated by cadherin switching leads to drug resistance in breast cancer. Decreased CDH1 expression is significantly correlated with a higher-grade, triple-negative receptor status, and poor prognosis in invasive breast carcinoma (89). Upregulation of cyclin D1, β-catenin, N-cadherin, MMP-2, MMP-9, and ICAM-1 and downregulation of CDH1 were found in doxorubicin-resistant TNBC cells (90). CDH1 is crucial for epithelial polarization and differentiation. Deregulation of CDH1 function plays a fundamental role in breast cancer metastasis and is associated with a worse prognosis (91). Over the past decade, many mechanisms have been identified to cause CDH1 inactivation in breast cancer (92, 93). In the 1990s, a correlation was found between the absence of CDH1 and the lobular subtype harboring CDH1 genetic alterations (94, 95). Subsequently, it is well-known that approximately half of the invasive lobular breast cancers show loss of heterozygosity, which is crucial for CDH1 dysfunction and loss of expression (93, 96). In addition, epigenetic alterations have emerged as a possible cause of aberrant CDH1 expression and function, such as hypermethylation of the CDH1 promoter site (97, 98). Abnormal glycosylation is also a potential cause of CDH1 dysfunction, because CDH1 is post-translationally modified by oxygen and nitrogen glycosylation (99, 100).

### Cadherins and EMT

**Figure 3:**
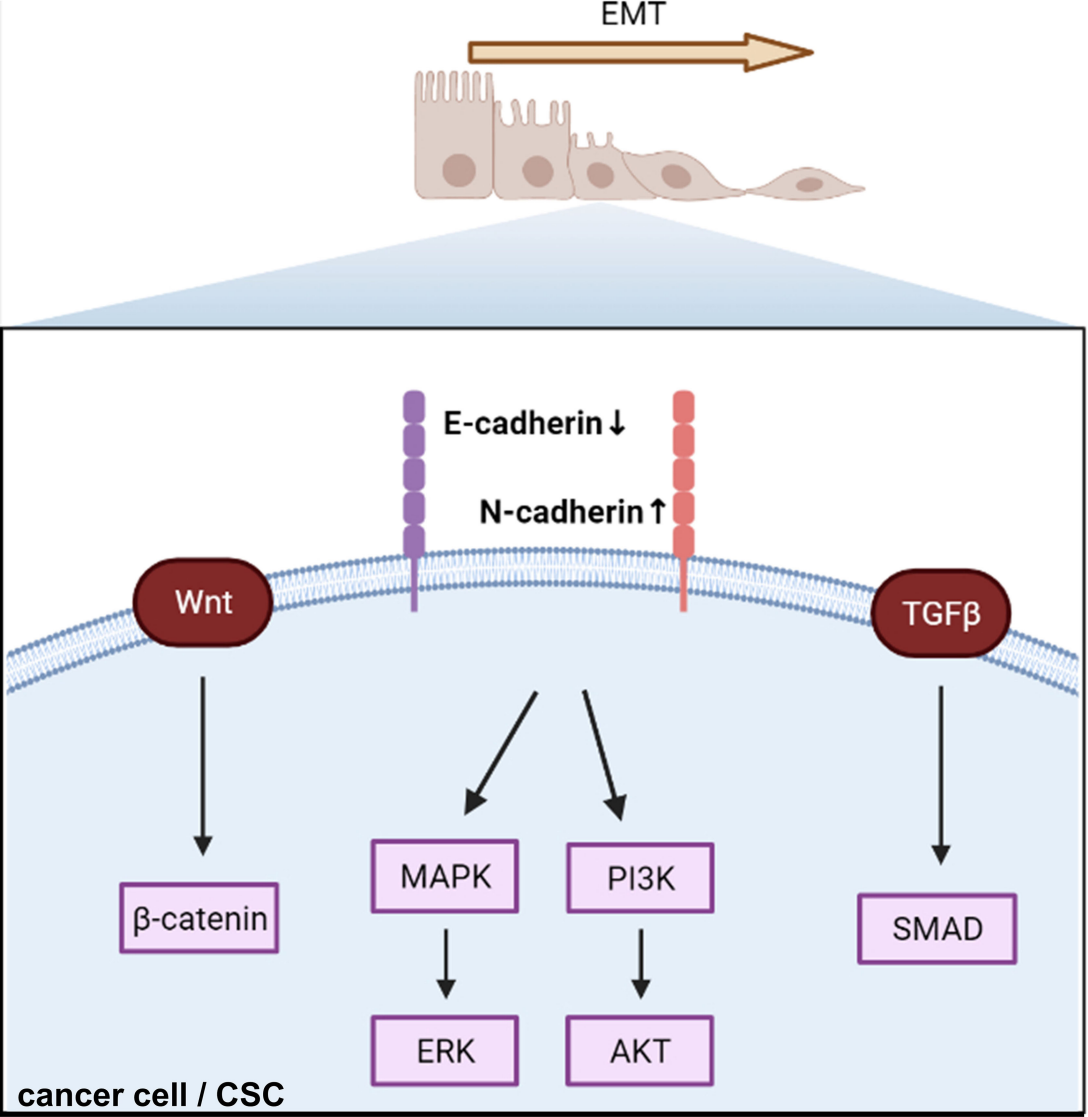
Cadherin regulates epithelial–mesenchymal transition and triggers intracellular signaling. EMT, epithelial–mesenchymal transition; CSC, cancer stem cell.

Many recent studies have focused on signaling changes in EMT in endocrine-related cancers (101–103). In this review, we have taken breast cancer as an example to illustrate recent EMT-related signaling (**Figure 3**). β-catenin, a critical cytoplasmic protein, confers cadherin-mediated cell-to-cell adhesion due to the transcriptional output of Wnt signaling, which leads to breast cancer tumor progression (104). Krüppel-like factor 9 (KLF9) blocks lung metastasis of breast cancer and increases CDH1 expression in 4T1 cells *in vivo*, indicating that the KLF9/CDH1 axis strongly contributes to breast cancer invasion and metastasis (105). N-cadherin-dependent cell-to-cell adhesion is required for migration mediated by bone marrow-derived MSCs towards MDA-MB-231 breast cancer cells *via* canonical TGF-β signaling (106). A previous study reported that aging breast ECM alone was sufficient to drive normal human mammary epithelial cells (KTB21) to an invasive and cancer-like phenotype that led to a loss of CDH1 *in vivo* (107). CDH1 facilitates metastasis in phenotypically sorted subpopulations of breast cancer cells, enabling clustering of circulating tumor cells (108). Snail exerts invasion, migration, and adhesion effects *via* regulation of N-cadherin and CDH1 in drug-resistant MCF-7/A cells (109). The authors also found that miR34a combined with doxorubicin suppressed the expression of Snail by inhibiting the Notch/NF-κB and RAS/RAF/MEK/ERK pathways in MCF-7/A cells (109).

### Cadherins and CAM-DR

Furthermore, the combination of miR34a and doxorubicin significantly repressed tumor growth in the MCF-7/A nude mouse xenograft model compared to doxorubicin alone (109). Cell motility was mediated by ERK1/2 and CDH1 was correlated with lactate supplementation (110). A study showed that activated ERK signaling is involved in radiotherapy-resistant breast cancer using MDA-MB-231 cells. CDH1/TGF-β/p-Smad2/3 signaling was found to be a survival axis in breast cancer by inhibiting reactive oxygen-dependent endogenous mitochondrial apoptosis (111). F-box and leucine-rich repeat protein 10 (FBXL10) interacting with SNAI1 promoted the migration and invasion of breast cancer cells by inhibiting CDH1 expression and inducing EMT (112). AKR1B10 downregulated CDH1 expression *via* the PI3K/AKT/NF-κB p65 signaling pathway in MCF-7 cells (113). Moreover, taxane therapy resistance was related to reduced PRP4K expression, depletion of PRP4K in TNBC cells (MDA-MB-231), and enhanced 2D migration, and 3D invasion correlated with higher fibronectin levels rather than changes in CDH1 (114). Connexins linked to N-cadherin, vimentin, Snail, and Zeb1 modulate CSC and EMT properties in breast cancer cells (90).

At the RNA level, overexpression of TPA diminished the expression of CDH1 and increased the expression of vimentin, fibronectin, and TGF-β1 (115). Circular RNA studies have shown that circNOLC1 (116) and circSCRIB (117) contribute to BC cell invasion and migration ability by reducing CDH1 and facilitating the tumorigenesis of breast cancer (118). Extracellular vesicles of insulin-like growth factor-1 (IGF-1), which is a crucial regulatory factor of mammary glands, promote the downregulation of CDH1 and upregulation of vimentin, N-cadherin, and MMP-9 in MDA-MB-231 breast cancer cells (119).

Interestingly, among environmental contaminants, including long-chain per- and polyfluoroalkyl substances (PFASs), a study has found that PFHxS can induce cell malignancy by reducing the levels of CDH1 and β-integrin and promoting cell migration and invasion in normal human breast epithelial cells (MCF-10A) (120).

Apart from CDH1 and N-cadherin, VE-cadherin promotes the attachment of breast cancer cells to the endothelial layer and initiates the incorporation phase instead of transmigration (121). As a result, cadherins mainly mediate CAM-DR *via* the EMT pathway and promote long-distance metastasis of breast cancer.

### Targeting of Cadherins

To date, there are few targeted therapies for cadherins. However, one potential practical strategy is the reversal of EMT by restoring CDH1 expression. For example, CTI-2 increased the expression level of CDH1 and decreased the expression levels of N-cadherin and vimentin through inactivation of the MAPK signaling pathway (122). MTDH, an miR-9-3p inhibitor, promotes the effect of gemcitabine on inducing apoptosis and inhibiting cell migration, invasion, and growth in breast cancer, suggesting that miR-9-3p regulated gemcitabine drug resistance (123). Interestingly, atorvastatin, an HMG-CoA reductase inhibitor, enhanced partial cancer-associated mesenchymal-to-epithelial reverting transition, and facilitated chemotherapy effects in metastatic TNBC (124). The sulforaphane-cisplatin combination restored CDH1 expression by altering chromatin modification and attenuated the metastatic potential of TNBCs by downregulating the SIRT-mediated EMT signaling axis (125). This study demonstrated that sulforaphane-cisplatin could overcome cisplatin resistance (125). The tumor-suppressive role of miR-205, suppressed by the CLDN11 gene, regulates EMT in breast cancer (126). Co-treatment with miR34a and doxorubicin slowed tumor growth in MCF-7/A cells *via* the Snail/CDH1 pathway *in vivo* (109). Intriguingly, many herbal or traditional medicines are available. JI017 in combination with paclitaxel can overcome paclitaxel resistance (127). Triptonide, a small molecule from the traditional Chinese medicinal herb, promotes the downregulation of N-cadherin, VE-cadherin, and VEGFR2 (128). Rhoifolin (RFL), a flavonoid from *C. nudiflora*, is a Chinese medicinal herb that affects CDH1, vimentin, Snail, and Slug, thereby eliciting anti-motile properties (129). Ethanol-based garlic extract prevents breast cancer evolution driven by a hypoxia-induced decrease in CDH1 and increased vimentin and motility (130).

## Selectins and Endocrine-Related Cancers

**Figure 4:**
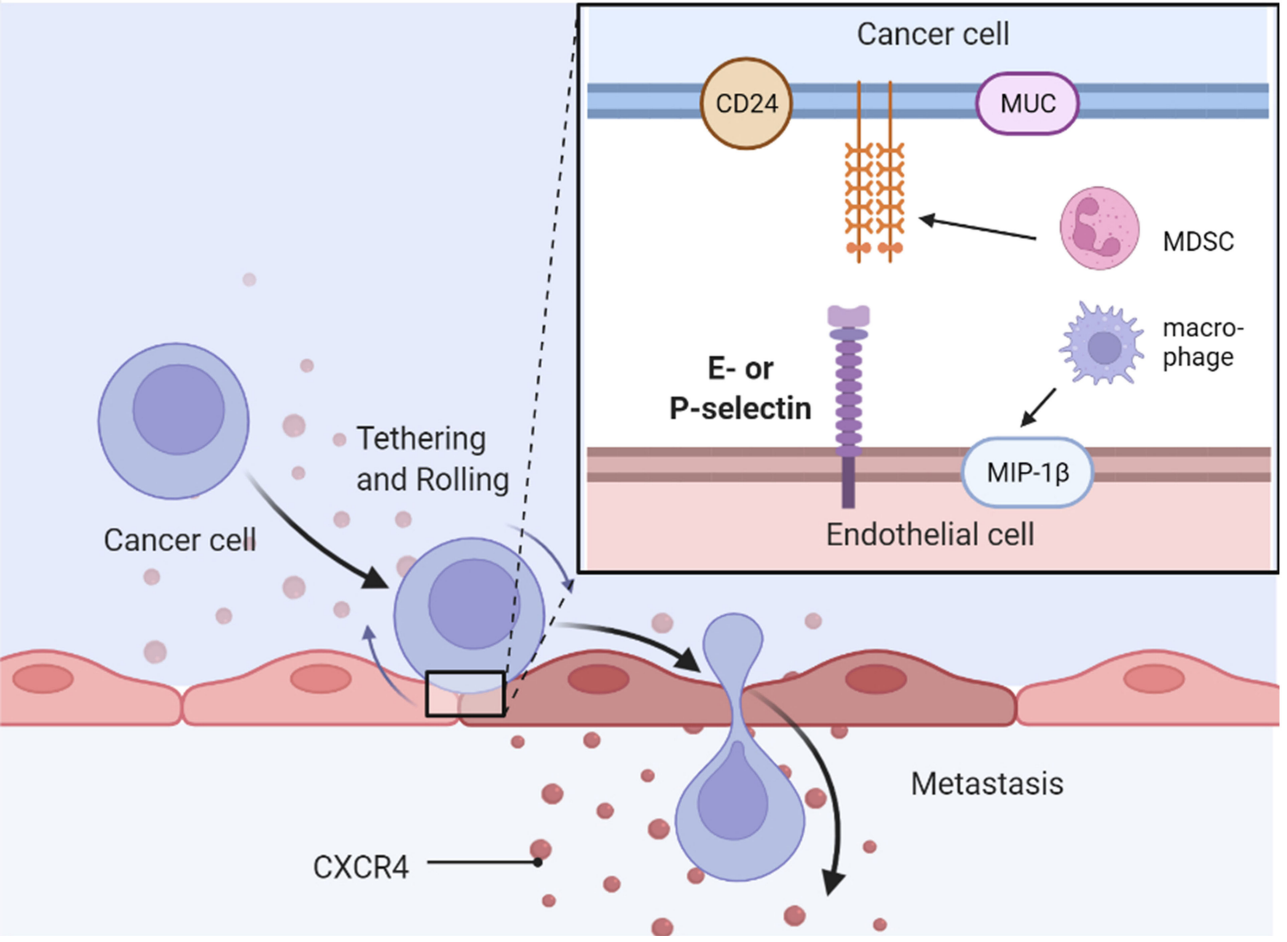
Selectins mediates metastasis of cancer cells. MDSC, myeloid-derived suppressor cell.

The role of selectins in cancer implantation and metastasis is well-established (131). Selectin ligands on cancer cells interact with selectins on endothelial or mesothelial cells, leading to extravasation of cancer cells into the blood, which is critical for hematogenous metastasis (132) (**Figure 4**). In addition to blood-borne metastasis, selectins mediate peritoneal dissemination in endocrine-related cancers (133). Platelets and leukocytes are also involved in metastasis *via* selective regulation (131, 134, 135). Selectins, particularly E-selectin, are involved in cancer drug resistance in acute myeloid leukemia (AML).

### Selectins and Their Ligands in Cancers

Inhibition of the interplay between selectins and their ligands has been studied as a potential strategy to provide therapeutic benefit in cancer. P-selectin glycoprotein ligand 1 (PSGL-1), a well-known selectin ligand, promotes the physiological and pathological progression of endocrine-related cancers (136). CD24 is another well-known P-selectin ligand that confers ovarian cancer progression by directly mediating ovarian cancer adhesion to tumor–mesothelial (49). MIP-1β secreted by alternatively activated macrophages elevated the expression of P-selectin on the mesothelial cell surface through CCR5/PI3K signaling, and ovarian cancer cells adhered to the *de novo* P-selectin *via* CD24 (49). MUC16 (CA125) is a selectin ligand expressed in metastatic pancreatic cancer cells (137) and epithelial ovarian cancer cells (138). Another mucin, called CA19-9, accelerates pancreatic cancer progression by binding to E-selectin, promoting angiogenesis, and regulating immunological response (139). Overexpression of MUC1, MUC9, and MUC13 has also been observed in ovarian cancer cells (140, 141). Two E-selectin ligands, BST-2 and LGALS3BP, are correlated with high liver and brain metastasis potential and poor survival in ER-negative breast cancer (142). Growth of pancreatic cancer cells was slowed down by E-selectin-dependent rolling and E-selectin-HA binding (143). Sialyl Lewis X on bone-metastatic prostate tumor cells exerted robust E-selectin binding activity by shear-flow manner (144). Contrarily, c-FOS, a negative regulator of E-selectin, significantly reduced ovarian cancer growth both *in vitro* and *in vivo* (48).

### Selectins and Metastasis

Breast cancer cells are anchored to the bone marrow microenvironment through the stromal-derived factor 1 (SDF-1)/E-selectin pathway (145). E-selectin also induces mesenchymal–epithelial transition and Wnt activation in cancer cells to promote bone metastasis (146). Furthermore, E-selectin plays a critical role in allowing breast cancer cells to infiltrate the bone marrow, and inhibition of the SDF-1/CXCR4 axis induces the mobilization of dormant cancer cells into circulation (147). Following lung-derived selectin inhibition, decreased lung migration and reduced proliferation were observed in TNBC through a 3D *ex vivo* pulmonary metastasis assay and lung-conditioned media in an *in vitro* model (148). In host mice, E- and P-selectin depletion inhibits intraperitoneal metastasis of PDA cells (149). P-selectin mediates platelet activation simultaneously in a combination of tissue factor-induced thrombin formation by pancreatic cancer cells (150) and breast cancer cells (151).

### Selectins and CAM-DR

Selectins, especially E-selectin, are also involved in cancer drug resistance in AML. E-selectin promotes endocrine-related cancer cell adhesion, chemotaxis, trans-endothelial migration, and stroma-induced drug resistance. Blocking E-selectin results in cancer cell mobilization from the TME to circulation and resensitizes cancer cells to chemotherapies (152). For example, uproleselan (GMI-1271), an E-selectin antagonist, received FDA approval for adult relapsed/refractory AML in 2017 and a phase 3 study is ongoing (153). A preclinical study demonstrated that AML cells have high E-selectin binding potential with chemotherapy resistance, resulting in a high leukemia relapse incidence. Chemoresistance of AML cells was resensitized to chemotherapy by both E-selectin host knockout (sele-/-) and uproleselan (154). GMI-1359, a dual CXCR4/E-selectin antagonist, reduced growth, enhanced docetaxel treatment, and restored docetaxel effectiveness in docetaxel-resistant prostate cancer cells (155). High expression of E-selectin with substantial CD45+ immune cell density adjacency was observed in doxorubicin-treated residual human breast tumors. Moreover, the functional blockade of E-selectin with an anti-E-selectin aptamer decreased the residual tumor burden and metastasis by inhibiting the TH2 shift (156).

Since L-selectin assists lymphocyte homing to lymph nodes, OX40L promotes effector T-cell expansion and inhibits regulatory T cells. A type of intelligent exosome engineered with L-selectin and OX40L was developed in a previous study (157). The exosome surface was functionally engineered with CD62L (L-selectin, a gene for lymphocyte homing to lymph nodes) and OX40L (CD134L, a gene for effector T-cell expansion and regulatory T-cell inhibition) by forced expression of the genes in the donor cells. These engineered smart exosomes were tested, and an improved outcome was observed in a breast cancer 4T1 xenograft model (157). As ovarian cancer cells can bind to L-selectin *in vitro*, RO-heparin, which has low anticoagulant activity, can inhibit L-selectin-mediated cell adhesion and prevent cancer metastasis (158).

Granulocytes are crucial factors in cancer metastasis. Cancer-induced expansion of immunosuppressive myeloid-derived suppressor cells (MDSCs) has been well described. Lu et al. first reported that PSGL-1 is expressed on the surface of MDSCs in pancreatic tumor tissues (159). They found that low-molecular-weight heparin (LMWH) could attenuate the early stage of adhesion between vascular ECs and MDSCs by inhibiting P-selectin/PSGL-1 binding. Therefore, fewer MDSCs are recruited to pancreatic tumor tissues (159). Furthermore, they reported that hydrophilic LMWH attenuated lung metastasis and alleviated the immunosuppressive TME through P-selectin blockage, thereby decreasing the recruitment of MDSCs to the lung (160). In addition to MDSCs, aged neutrophils regulate metastasis (161). Compared to CXCR4 low/CD62L high immature neutrophils, aged neutrophils facilitate breast cancer migration and mediate metastasis through increased release of neutrophil extracellular traps (161).

Fucoidan, a sulfated polysaccharide, has shown a strong affinity towards P-selectin and is widely used for its anticoagulant, antitumor, and anti-inflammatory effects; therefore, fucoidan-Dox NPs (FU-Dox NPs) were developed. The MDA-MB-231 cell line with high P-selectin expression is more sensitive to FU-Dox NPs than the MDA-MB-468 cell line that exerts low P-selectin expression (162). Interestingly, P-selectin-mediated adhesion is upregulated by sulfatide in a breast cancer model. However, increased sulfatide synthesis sensitizes cancer cells to hypoxia and doxorubicin (163).

## Integrin and Endocrine-Related Cancers

**Figure 5:**
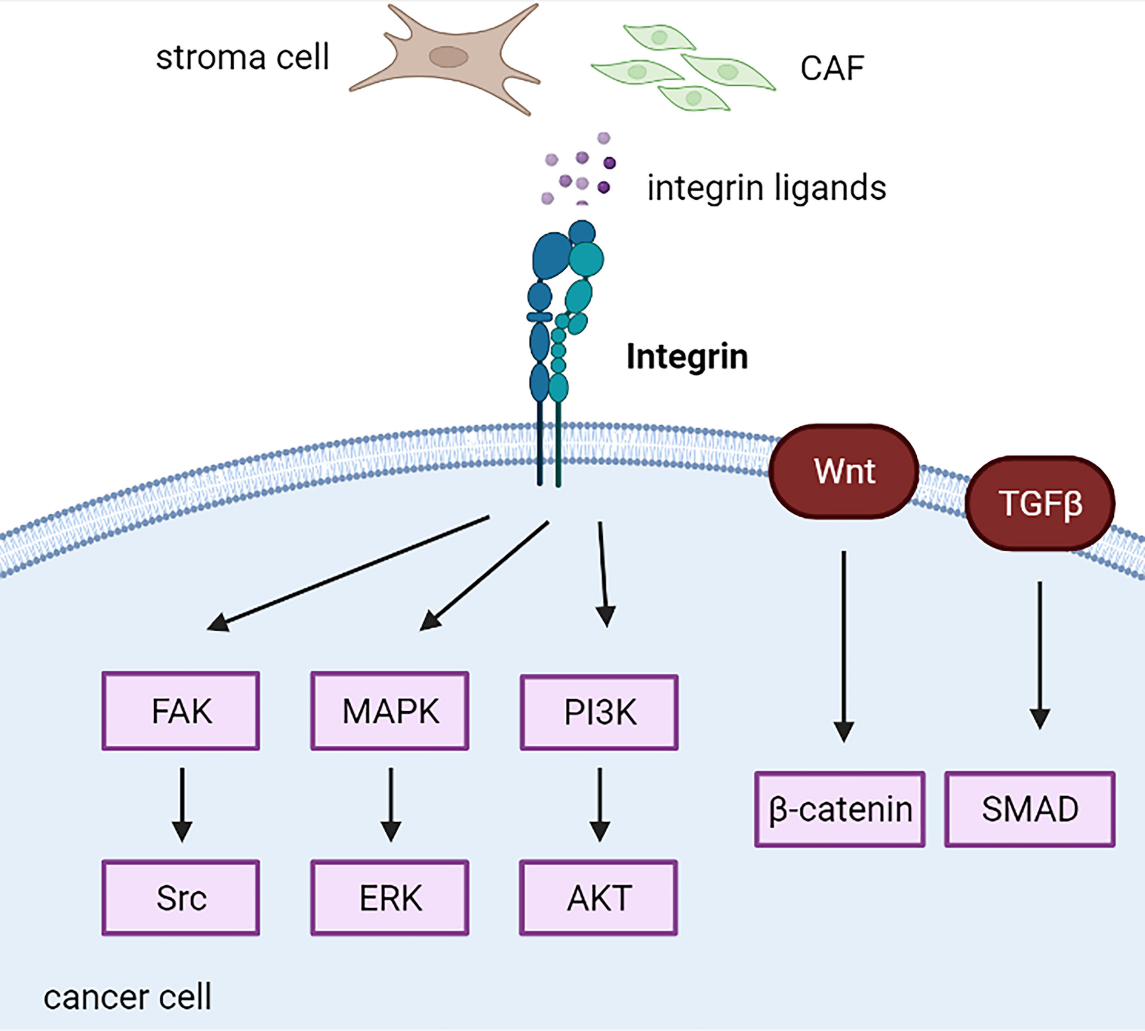
Integrin-related cascades in cancer cells. CAF, cancer-associated fibroblast; CSC, cancer stem cell.

According to their bidirectional signaling character, integrins present two conformational states that determine the receptor affinity of ECM proteins: a bent integrin (inactive form) shows a low affinity for ECM ligands, whereas an extended integrin (active form) elicits the activation of downstream signaling (**Figure 1A**). Abnormal regulation of integrins impacts cancer cell and stroma crosstalk, maintains cancer stemness characteristics, facilitates the formation of metastases, and induces drug resistance (164, 165) (**Figure 5**). For example, Kim et al. systemically demonstrated integrin α4 and α6 mediated drug resistance in B-cell acute lymphoblastic leukemia, and either integrin α4 or α6 inhibition led to increased chemosensitivity (166–168).

Increased integrin expression mainly indicates poor outcomes in endocrine-related cancers. For example, integrin α3 is highly correlated with unfavorable prognosis in papillary thyroid carcinomas (169). Increased expression of integrins α5 and β1 is associated with the development of chemotherapy resistance and is an independent predictor of worse overall survival in epithelial ovarian cancer (170). Increased expression of integrin αV in PDAC cells has been associated with decreased patient survival (149). High expression of integrin α2β1 co-expressing HGFR or CD44v6 indicated worse progression-free survival in primary ovarian cancer and supported the hypothesis of mediating platinum resistance mediated by integrin α2β1 (171). Oxysterol-binding protein-like (OSBPL) family members potentially bound to integrins are highly upregulated in PDAC with poor outcomes in pancreatic ductal adenocarcinoma PDAC (172). Furthermore, increased integrin β1/lnc005620 regulated chemoresistance to epirubicin in TNBC *in vitro* and *in vivo* (139). However, integrin α3 is a favorable prognostic biomarker in breast cancer *via* integrin α3-ECM mediated immune cell infiltration (173). Increased integrin α5 expression induces lower aggressiveness in tamoxifen-resistant breast cancer (174).

### Integrins and CSCs

Several studies have implicated integrins as vital regulators of CSCs. Integrin β1, specifically integrin α3β1, is necessary for mammary tumorigenesis (175–177). Apart from stemness, integrin β1 promotes proliferation by increasing the expression of TGF-β receptor 2 and kindlin-2 signaling in the MIA PaCa-2 pancreatic cancer cell line (178). Knockout of integrin β1 decreased the expression of integrin α5, which resulted in impaired cell metastasis and adhesion to vitronectin and fibronectin (178). Integrin α5β1-targeted micellar paclitaxel (ATN-MPTX) successfully selectively inhibited immunogenic cell death using chemo-immunotherapy in TNBC cells (132). Interestingly, downregulation of integrin β1 is compensated for by increased β3 integrin expression (179).

Integrin β3 regulates breast cancer stemness through the Wnt/β-catenin/HOXD3 pathway and mediates drug resistance (180, 181). In addition, integrin αvβ3 is a potential marker of breast and pancreatic cancers with stem-like properties and high resistance to receptor tyrosine kinase inhibitors, such as erlotinib and other chemotherapies, through the KRAS-RalB-NF-κB pathway and the mTOR/mTORC1 axis (182, 183). Moreover, integrin αvβ3 mediated PARP inhibitors’ resistance in breast cancer cells *via* the TGFBI-ZEB1 pathway using the CRISPR deletion method (184). Furthermore, TGFBI produced by macrophages during immunosuppression in early ovarian cancer cells may be an effector of the tumor-promoting factor TGF-β (185). Integrin αv activates latent TGF-β and drives EMT in PDAC cells (149). Similarly, integrin α6 induced EMT with the upregulation of N-cadherin and downregulation of CDH1 in ovarian cancer spheroids *via* the TGF-β1/Smad3 pathway (186). Interestingly, TGF-β1 promotes the expression of SMYD3 and ITGB6 as feedback mechanisms (186). Integrin α6 is enriched and enhances stem cell phenotypes in breast CSCs, especially in TNBC cells (187, 188). Integrin α2, a direct target of miR-206, promotes TNBC self-renewal-related mammosphere formation, where canonical Wnt signaling is involved (189). Recently, a study demonstrated that upregulation of integrin β8 mediated by lnc-TCF7 leads to ovarian cancer stemness (190). Integrin αvβ8, a key activator of TGF-β, regulates immunotherapy and radiotherapy resistance, which may be reversed by a potent blocking monoclonal antibody against integrin αvβ8 (ADWA-11) in prostate cancer (191). The anti-integrin β8 antibody treatment mechanism restores the *in vitro* cytotoxic effect of tumor CD8 T cells by inhibiting Treg cells (191, 192).

### Integrins and FAK/Src/PI3K Axis

FAK is a downstream protein that is highly phosphorylated in response to integrin activation and contributes to cancer cell behaviors, including resistance to anoikis and maintenance of an immunosuppressive TME (193, 194). Therefore, FAK inhibition increased immune surveillance in immunosuppressive pancreatic ductal adenocarcinoma TME and restored tumor response to immunotherapy, such as PD-1 antagonists (194). Integrin β1/LRRC15 exerts a metastatic invasion role *via* activation of FAK in patient-derived ovarian cancer xenograft models, inhibited by ABBV-085, an antibody–drug conjugate of LRRC15 (195). Following binding to integrin, PCMT1 was released from ovarian cancer cells, leading to activated FAK-Src signaling to promote cancer progression and anoikis resistance (196). Moreover, stiffness-induced autophagy in stromal cells mediated by integrin αV-PTK-AMPKα promotes the growth of adjacent pancreatic stellate cells *in vitro* and *in vivo* (197). Integrins α3 and β5 were increased in highly metastatic breast cancer cell lines, such as MDA-MB-231 and MDA-MB-231BO, while decreased in poorly metastatic MCF-7 cells. Knockdown of integrin α3 and β5 leads to the inhibition of migration and invasion of breast cancer cells through the FAK/Src/Rac1 pathway (198). Increased integrin β1 contributes to resistance to anti-HER2 (trastuzumab or lapatinib) and anti-PI3K (pertuzumab) *via* the activation of FAK/Src signaling in HER2-positive breast cancer (199, 200).

Integrins also play an essential role in stimulating the activity of Ras, which in turn activates the MAPK/ERK and PI3K/Akt signaling pathways (201, 202). Both the MAPK/ERK and PI3K/Akt signaling pathways are critical cascades that regulate cancer cell proliferation, survival, and drug resistance, which have been well investigated in the past decades (203). CAF-derived THBS2 bound to integrin αvβ3 promotes cancer cell growth and adhesion *via* the MAPK pathway in PDAC cells *in vitro* and *in vivo* (204). In addition, serial studies have implicated that inhibition of integrins efficiently sensitizes breast cancer cells to conventional therapies, such as radiotherapy. Integrin β1 inhibition by AIIB2 in combination with post-ionizing radiation increases apoptosis compared with radiation alone by downregulating Akt in breast cancer (205). Integrin α6 mediates radiation resistance *via* PI3K/Akt and MEK/ERK signaling (206). Therefore, the PI3K inhibitor LY294002 and MEK inhibitor U0126 reverse radiation resistance was mediated by integrin α6 in breast cancer cells (206). Conversely, integrin α6β1 mediates resistance to PI3K inhibitors in PTEN-negative prostate cancer by inducing PIM kinases and oxidative stress (207). Recently, the upregulation of IGF1R-integrin α6-S100A4 signaling was found to promote chemoresistance to metastasis of epithelial ovarian cancer cells (208). S100A4 is secreted from lung fibroblasts and can reciprocally activate them (208). Integrin β1 is regulated by cysteine-rich angiogenic inducer 61 (CYR61), a matricellular protein, in TNBC (209). In addition, CYR61 integrin β1-AMPKα mediates lung metastasis, anoikis resistance, and extravasation of TNBC cells in an AKT-, FAK-, and ERK1/2-independent manner (209).

## Outlook

**Figure 6:**
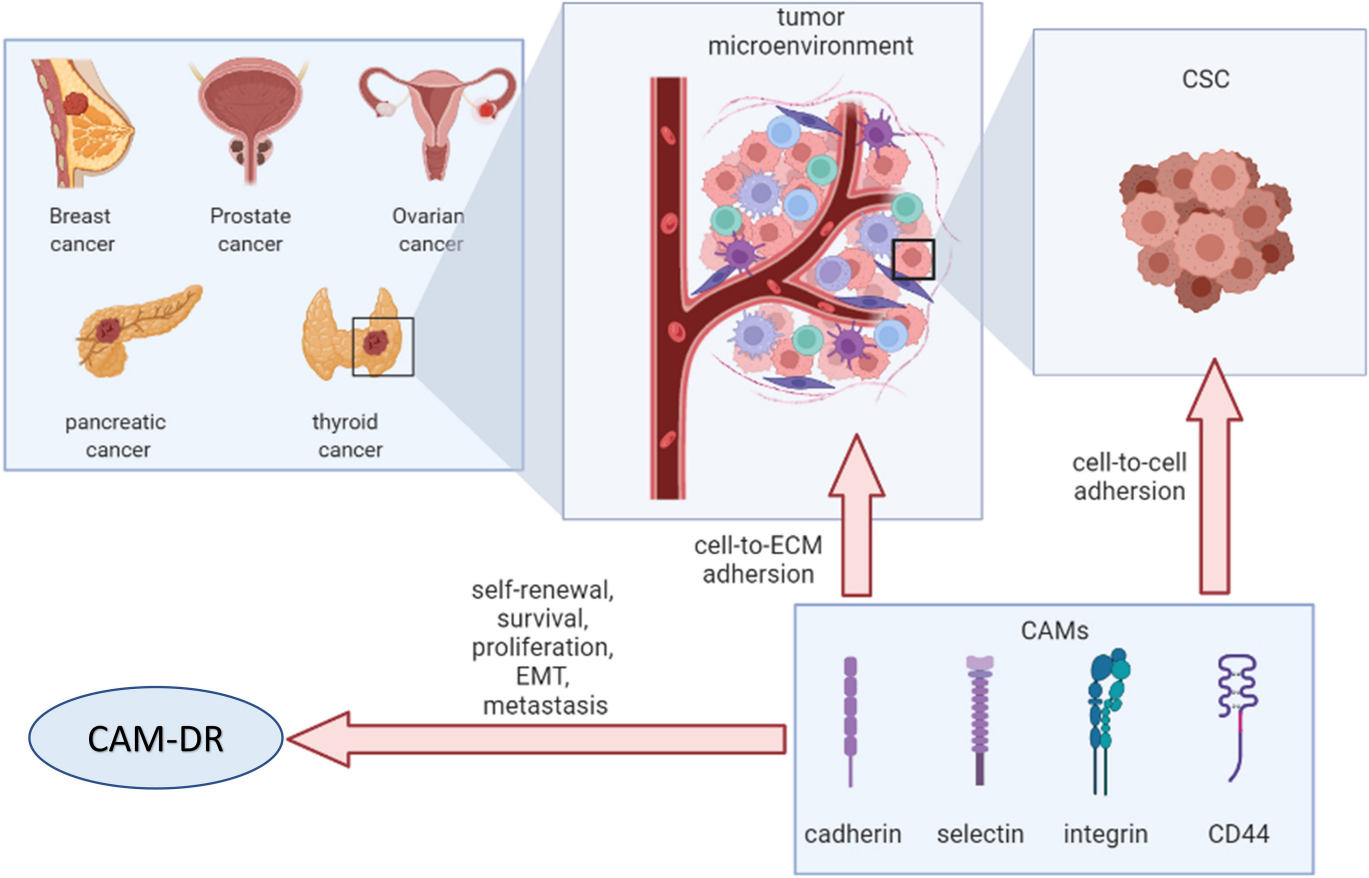
Overview of cell adhesion molecules in endocrine-related cancers. CSC, cancer stem cell; ECM, extracellular matrix; CAMs, cell adhesion molecules; EMT, epithelial–mesenchymal transition; CAM-DR, cell adhesion-mediated drug resistance.

CAMs contribute to the attachment of cancer cells to the TME *via* cell-to-cell and cell-to-ECM interactions. Remodeled TME mediates drug resistance in cancer cells, resulting in altered expression of CAMs (**Figure 6**). Therefore, CAMs may be an attractive therapeutic target for cancer intervention. Further mechanisms of CAM-DR in endocrine-related cancers are warranted to open new avenues for more effective treatments in the future.

## Author Contributions

YR conceptualized the study. YR, LC, and DX wrote the original draft. YR, LC, DX, XF, and XW wrote, reviewed, and edited the manuscript. YR and XW supervised the study and YR acquired funding. All authors have contributed to the manuscript and approved the submitted version.

## Funding

This study was supported by the GuangDong Basic and Applied Basic Research Foundation (Grant No.2022A1515010012), the President Foundation of Nanfang Hospital, Southern Medical University (Grant No. 2020C003), and the State Scholarship Fund from the China Scholarship Council (File No. 201809315008).

## Conflict of Interest

The authors declare that the research was conducted in the absence of any commercial or financial relationships that could be construed as a potential conflict of interest.

## Publisher’s Note

All claims expressed in this article are solely those of the authors and do not necessarily represent those of their affiliated organizations, or those of the publisher, the editors and the reviewers. Any product that may be evaluated in this article, or claim that may be made by its manufacturer, is not guaranteed or endorsed by the publisher.
